# Periodontal Health and Its Relationship with Psychological Stress: A Cross-Sectional Study

**DOI:** 10.3390/jcm13102942

**Published:** 2024-05-16

**Authors:** Monica Macrì, Giuseppe D’Albis, Vincenzo D’Albis, Anna Antonacci, Antonia Abbinante, Riccardo Stefanelli, Francesco Pegreffi, Felice Festa

**Affiliations:** 1 Department of Innovative Technologies in Medicine & Dentistry, University “G. D’Annunzio” of Chieti-Pescara, 66100 Chieti, Italy; 2 Department of Interdisciplinary Medicine, University of Bari “Aldo Moro”, 70121 Bari, Italy; 3 Department for Life Quality Studies, University of Bologna, 40064 Bologna, Italy; 4 Department of Biomolecular Sciences, University of Urbino Carlo Bo, 61029 Urbino, Italy

**Keywords:** periodontal health, perceived stress, mindfulness, bleeding index, periodontitis

## Abstract

**Background**: Studies suggest that chronic psychological stress can lead to oral health deterioration, alter the immune response, and possibly contribute to increased inflammation, which can impact the physiological healing of periodontal tissues. This cross-sectional study seeks to assess and improve clinical understanding regarding the relationship between perceived stress, mindfulness, and periodontal health. **Methods**: A total of 203 people were analyzed from December 2022 to June 2023. The Periodontal Screening and Recording (PSR) score and Gingival Bleeding Index (GBI), and Plaque Control Record (PCR) of every patient were registered. Subsequently, participants completed the Sheldon Cohen Perceived Stress Scale (PSS) and the Mindfulness Awareness Attention Scale (MAAS) questionnaires. The collected data underwent statistical analysis, encompassing the evaluation of correlations and dependencies. Applying Welch’s *t*-test to assess the relationship between MAAS and the variable indicating the presence or absence of periodontitis, a noteworthy *p*-value of 0.004265 was obtained. **Results**: This underscores a significant distinction in MAAS scores between patients affected by periodontitis and those unaffected by the condition. Additionally, Pearson correlations were computed for GBI and perceived stress, PCR and perceived stress, PCR and MAAS. The resulting *p*-values of 2.2–16, 3.925–8, and 2.468–8, respectively, indicate a statistically significant correlation in each instance. **Conclusions**: These findings contribute valuable insights into the interconnectedness of these variables, emphasizing the significance of their associations in the study context. Despite the limitations, the findings of this study suggest a significant relationship between psychological stress, mindfulness, and periodontal tissue health. Clinical trials are necessary to incorporate the assessment of a patient’s psychological status as a new valuable tool in the management of periodontal health.

## 1. Introduction

Periodontitis is a multifactorial chronic inflammatory disease characterized by the progressive destruction of the tooth-supporting apparatus. It involves clinical attachment loss (CAL) [1], radiographic assessment of alveolar bone loss [2], periodontal pockets [3], and gingival bleeding [4].

Periodontitis is linked to specific bacterial species, with lifestyle factors, smoking, diabetes, genetic anomalies, and stress influencing its course. It is estimated that 60% of the population is affected by periodontitis, with 10–14% suffering from severe forms (stages III and IV) [5,6].

The dentist establishes a conclusive diagnosis of periodontal disease by documenting clinical biometric parameters such as pocket depth, recession, total attachment loss, furcation lesions, and mobility. A radiological examination evaluates the periodontium, including crestal bone, lamina dura, and root morphology [7,8]. Periodontal disease progression is monitored with radiographic exams [9].

It is proven that while bacteria are indispensable, they are insufficient for periodontal disease to manifest. A susceptible host, environmental factors, and a specific microbiome are key elements for active periodontal disease [10]. Heredity plays a role, especially in patients with early-onset periodontitis [11]. Host-related risk factors include diabetes, HIV infection, and smoking [12,13]. Smoking, poor oral hygiene, and plaque-retention conditions are environmental risk factors that aggravate the disease. Smoking also promotes pathogenic bacteria accumulation [14].

Risk factors directly increase the likelihood and progression of periodontal disease. They cause alterations in the subgingival microbiota, affecting disease progression and response to treatment [15].

Staging is the snapshot of the patient’s condition, providing a severity index of the disease based on interproximal attachment loss, CAL/PAC, and Radiographic Bone Loss (RBL). It indicates the extent and distribution of dental compromise, introducing the complexity of managing and treating the individual patient.

### 1.1. Psychological Aspects in Patients with Periodontitis

Hans Selye introduced the concept of stress in 1936 as the “General Adaptation Syndrome” [16], describing the body’s response to prolonged exposure to various stressors—physical (e.g., fatigue), mental (e.g., work demands), and social or environmental stimuli (e.g., social obligations) [17]. Stress can lead to eustress (beneficial) or distress (harmful), involving physiological and pathological consequences. The General Adaptation Syndrome (GAS) consists of three stages: alarm, resistance, and exhaustion/recovery, each with distinct physiological responses [18].

Recovery occurs when the body eliminates the effects of the stressor or the stressor itself [19]. The sequence involves exposure or anticipation of stressors, reception, processing, and executing the adaptation syndrome, leading to modifications in various homeostatic systems [20,21].

Stress is a risk factor for periodontitis and other oral diseases, as it can alter the immune response, increasing susceptibility to bacterial dysbiosis. Stress linked to periodontitis falls into three categories: micro-stress from everyday tension, significant life events like divorce, and emergencies or disasters. These stressors are associated with compromised periodontal health and disease progression. Stress can also lead to bruxism and teeth clenching [22].

Cortisol, a hormone linked to stress, has detrimental effects on periodontal health. Produced by the adrenal gland, it can impair immune competence and increase blood glucose levels, raising susceptibility to periodontal disease. People with periodontitis and high stress levels often exhibit elevated cortisol in blood serum and saliva. Although not common in dental practice, cortisol measurement could become a tool for assessing stress [23].

Psychological stress can affect periodontal health through behavioral changes, leading to neglected oral hygiene, increased smoking, alcohol consumption, and poor dietary choices. Risk factors associated with stress and periodontal disease include insufficient sleep, inadequate nutrition, poor oral hygiene practices, smoking, and recent illnesses [24]. These factors, coupled with plaque buildup, contribute to altered body responses and compromised periodontal health.

### 1.2. Mindfulness and Behavior Change

Mindfulness, stemming from the Pali word ‘sati’, refers to intentional awareness sustained with effort. It is both a psychological technique and an approach in cognitive and behavioral psychology, focusing on present-moment awareness and re-centering attention when distracted. It involves observing the mind without attachment to its contents, such as sensations and thoughts, promoting intuitive self-knowledge and cognitive distancing from thoughts. This approach aims to reduce suffering by changing one’s relationship with mental content [25].

This change facilitates a flexible ability to detach from mental contents, allowing them to be observed more clearly. This detached mindfulness [26] reduces automatic reactivity, which typically leads people to seek rapid ways to avoid suffering. Ironically, these efforts can cause additional suffering due to unrealistic emotional ideals, resistance to the present moment, and happiness goals set in the future. Mindfulness encourages acceptance of the present, a broader understanding of difficulties, and tolerance of negative emotions.

Empirical studies demonstrate that regular meditative practice affects key brain structures involved in emotion regulation and metabolic activity in the body [27]. These changes reflect positive alterations in brain function, with measurable benefits in blood parameters, showing results beyond those achieved with other relaxation techniques.

Notable effects include increased activity in the left prefrontal cortex, associated with positive emotions, and in the right hemisphere’s deep nuclei, linked to intuitive aspects of perception. There is also a remodulation of activity in the amygdala, related to fear. The intervention is neuro-modulated by cytokines, other neuromodulators, and glucocorticoids, affecting the hypothalamic–pituitary axis and cortisol secretion. Mindfulness also reduces inflammatory markers like interleukin-6and adapts cell-mediated immunity, potentially leading to protective mechanisms on DNA through increased telomerase activity. These changes are linked to reduced signs of aging at both cellular and brain levels, along with improved outcomes in some disabling conditions [28,29,30,31,32,33].

These effects extend to psychological benefits like enhanced stress management, reduced depressive relapses, better anxiety control, increased creativity, and heightened feelings of happiness [34].

The study and application of mindfulness intersect psychology, neuroscience, and medical fields, including behavioral medicine, rehabilitation, gynecology, oncology, endocrinology, cardiology, immunology, algology, and psychosomatic medicine.

### 1.3. Psychology and Oral Health

Respected research groups worldwide have extensively studied the emotional and psychological influences on oral and periodontal health. A cohort study of 621 individuals found a significant link between stress levels and the prevalence of periodontal issues. Using the Perceived Stress Scale (PSS), researchers found that those with higher stress exhibited a 15–36% greater incidence of periodontal problems. Another study analyzed stress-coping strategies in chronic periodontitis patients, highlighting a connection between inadequate stress management, especially defensive strategies, and a higher risk of severe periodontal disease. Additionally, a study with 66 patients examined the impact of stress and anxiety on the effectiveness of non-surgical periodontal treatment. Using the Stress Symptoms Inventory (SSI) and the Spielberger State–Trait Anxiety Inventory (STAI), researchers found correlations between anxiety, stress traits, and treatment outcomes, indicating that emotional factors can affect periodontal intervention success [35,36,37].

These studies contribute to understanding the complex relationship between psychological factors and oral health, providing insights beyond the traditional scope of periodontology research. Our objective is to add new data to enhance our understanding of these dynamics, aiming to shed light on the complex interactions identified in earlier research.

Our study assesses the complex interplay among periodontal health, stress, and mindfulness. Building on previous studies, we analyzed comprehensive periodontal data and conducted attitudinal tests to gauge participants’ perceptions of stress and mindfulness. To carry out this assessment, a statistical analysis was conducted on the periodontal clinical data and patient-reported psychological data.

## 2. Materials and Methods

The study was conducted between January and June 2023 with Italian guidelines. The study protocol was in accordance with the Declaration of Helsinki of 1965, revised in Tokyo in 2004. It was registered in the National Library of Medicine and National Center for Biotechnology Information with the identifier code NCT05849415, approval date: 23 April 2023.

### 2.1. Participants

Patients referred to the Department of Dentistry of the Hospital of Bari were invited to participate in the study voluntarily. Before the commencement of the study, all participants were given an information letter outlining the study procedure, data evaluation, and data protection measures. To ensure anonymity, each participant was assigned an ID number.

The only inclusion criterion was being at least 18 years old. The exclusion criteria were:

- Pregnancy.
- Administration of antibiotics in the last 15 days before entry into the study or indications for antibiotic prophylaxis.
- Orthodontic appliances.
- Immunological diseases or use of drugs that can affect oral tissues (phenytoin, cyclosporine), nifedipine, chronic use of nonsteroidal anti-inflammatory drugs.
- Refusal to participate in the study.
- All patients with systemic conditions that could potentially impact periodontal health (individuals with conditions such as high blood pressure, diabetes, and chronic nephrosis).

The participants’ general condition and dental history were evaluated by medical interview (these data were self-reported by the subjects).

### 2.2. Periodontal Examination

**Figure 1:**
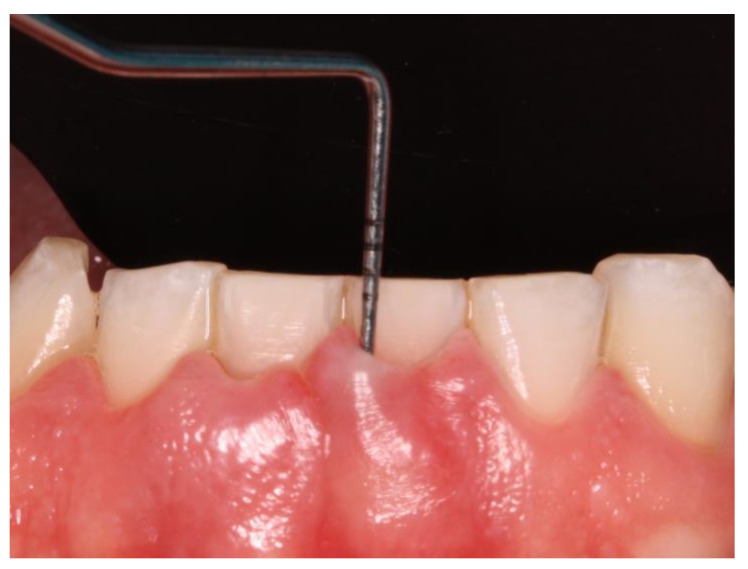
Periodontal probing.

Clinical data of all teeth, excluding third molars, were obtained during oral examinations at initial visits. Accredited Oral Hygienists and Dentists performed whole-mouth oral examinations. The manual periodontal probe PCPUNC15 was used for oral examinations. Oral examinations included periodontal pocket depth (PPD; 6 sites per tooth) (Figure 1) and bleeding on probing (GBI; 4 sites per tooth) [38].

These data were collected in a periodontal charting, and the Periodontal Screening and Recording (PSR) score and Gingival Bleeding Index (GBI) were calculated.

The mouth has been divided into sextants or sections—right maxillary, left maxillary, anterior mandibular, right mandibular, left mandibular, and posterior mandibular. The highest score of the following has been recorded for each tooth in each sextant:

- 0 for healthy periodontium;
- 1 for bleeding upon probing or dental calculus;
- 2 for pocket depth less than or equal to 3.5 mm;
- 3 for pocket depth greater than 3.5 mm and less than or equal to 5.5 mm;
- 4 for pocket depth greater than 5.5 mm;
- X for tooth/teeth missing or excluded from recording.

The scores for each sextant have been added up and divided by the number of teeth in that sextant to obtain the sextant score. The overall PSR score has been calculated by taking the highest sextant score [39].

**Figure 2:**
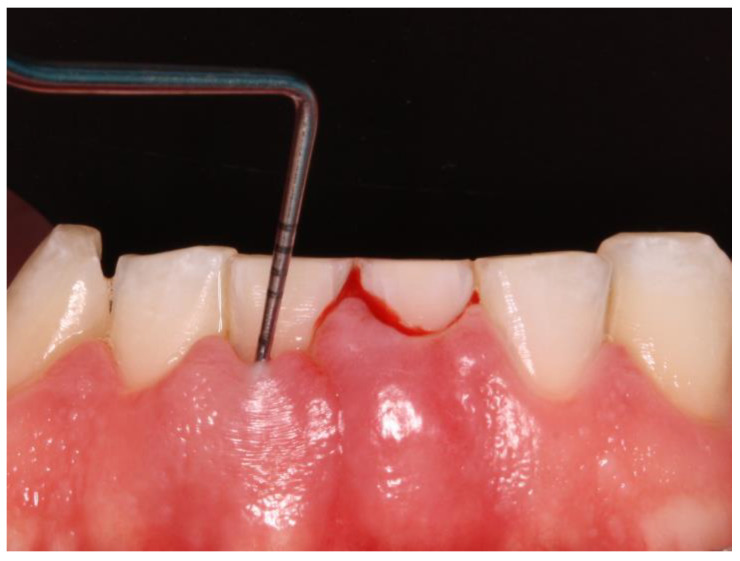
Bleeding after periodontal probing.

The Gingival Bleeding Index (GBI) was performed to assess the presence or absence of gingival inflammation. Bleeding areas were determined by running the periodontal probe over four points per tooth (mesial, distal, buccal, and lingual) (Figure 2). The calculation to obtain the percentage value was as follows: number of bleeding sites/number of evaluated sites ×100.

After careful assessment, it was recorded whether each patient was affected by periodontal disease (1) or not (0).

### 2.3. Perceived Stress Scale and Mindfulness Awareness Attention Scale (Self-Report)

Subsequently, each patient completed 2 attitudinal and evaluation tests in paper or digital format. The first attitudinal test completed was the “Perceived Stress Scale”, or PSS, which evaluates the subjective perception of stress. The questionnaire consists of 10 questions regarding feelings and thoughts during the last month. Possible answers range from 0 to 4 based on the severity or frequency with which the person has thought in a certain way. For each question, the alternatives are as follows: 0—never, 1—rarely, 2—sometimes, 3—reasonably often, 4—very often. Therefore, the total score can range from 0 to 40. The range between 0 and 13 represents a perceived stress value that is either nonexistent or mild and well managed. The range between 14 and 26 represents a moderate stress value that can negatively affect physical and mental health. The range between 27 and 40 represents a severe perceived stress value that significantly compromises physical and mental health.

The second test administered to the patients was the Mindfulness Awareness Attention Scale, which measures an individual’s tendency towards intentional awareness based on the frequency with which they are either mind-full or mind-less in their experiences. To measure the scale, one must calculate the average of the 15 responses. Higher scores reflect higher levels of disposition towards mindfulness.

The entire dataset has undergone rigorous analysis and scrutiny conducted by seasoned experts in the field of mathematical statistics. Proficient professionals meticulously examined the data with a profound understanding of mathematical modelling, statistical methods, and analytical techniques.

The following basic statistics have been calculated for the variables: Age, PCR, GBI, PSR, PSS, and MAAS. The occurrences for the variables “Gender” and “Periodontitis” were then calculated. The Poisson regression analysis was performed with the variable “Periodontitis” as the dependent variable, and the variables “GBI”, “Age”, “Perceived stress”, and “PCR” were included as predictors in the model. A difference test between two groups (0 and 1) was then performed for the numeric variable MAAS, which was divided based on the variable periodontitis (presence or absence of periodontitis). A linear regression analysis was then performed using the lm model in R. The Pearson Chi-square test was finally conducted to evaluate the association between the two categorical variables “Perceived Stress” and “MAAS” in the dataset.

In the analysis described in the present article, the psychological evaluation was performed using two tests compared to the mentioned articles. The first one was the Perceived Stress Scale. PSS is a widely used measure designed to assess perceptions of recent stress. A questionnaire is used in various fields of medicine to evaluate the relationship between stressful, emotional states and other medical conditions [40,41,42,43,44].

The second test used was the MAAS. Researchers consider the MAAS a reliable scale of awareness with high reliability and a strong correlation with other related constructs such as meditation, rumination, and self-awareness, as well as a genuine measurement of mindfulness [45].

The MAAS is a 15-item scale designed to assess a fundamental characteristic of mindfulness, which is open or receptive awareness in bringing attention to what is happening in the here and now. The MAAS scale shows strong psychometric properties and has been validated by the scientific community in the psychiatric and psychological field and in patients with oncological pathologies [46,47,48]. Clinical evaluations and laboratory studies have shown that MAAS detects a unique quality of consciousness associated with and predictive of a range of self-regulation and well-being constructs [49].

### 2.4. Statistical Analysis

We used Poisson regression to analyze count data. In particular, the relationship between Age and Perceived Stress, Gender and Psychological Score, Periodontitis and GBI, Periodontitis and PSS, GBI and PSS, GBI and MAAS, PCR and PSS, and PCR and MAAS was analyzed. Appropriate for variables that follow a Poisson distribution with non-negative integer values and equal mean and variance, this method predicts the occurrence of the dependent variable based on independent variables, using maximum likelihood estimation [50]. The model employs a natural logarithm link function, assuming a linear relationship between the log of the rate parameter and the independent variables. The estimated coefficients indicate the impact of independent variables on the expected count or rate of occurrence [51].

Additionally, we used the *t*-test to identify significant differences between group means, suitable for normally distributed data with equal variances. If these conditions were not met, we used Welch’s *t*-test, which is more robust to unequal variances. Welch’s *t*-test calculates the t-value by dividing the difference between group means by the standard error, considering sample sizes and variances. Its degrees of freedom are calculated using a more complex formula, with hypothesis testing and interpretation similar to the traditional *t*-test [52].

The association between periodontal values and the MAAS and Perceived Stress test was carried out using this method.

## 3. Results

The resultant participation rate was 100%. All participants agreed to undergo periodontal screening and consented to answer the two written questionnaires.

The dataset contains 203 observations and 8 variables. The following basic statistics have been calculated for the variables (Table 1).

The occurrences for the variables “Gender” and “Periodontitis” respectively show 96 females and 107 males, and for periodontitis, 57 negative responses (0) and 146 positive responses (1).

**Figure 3:**
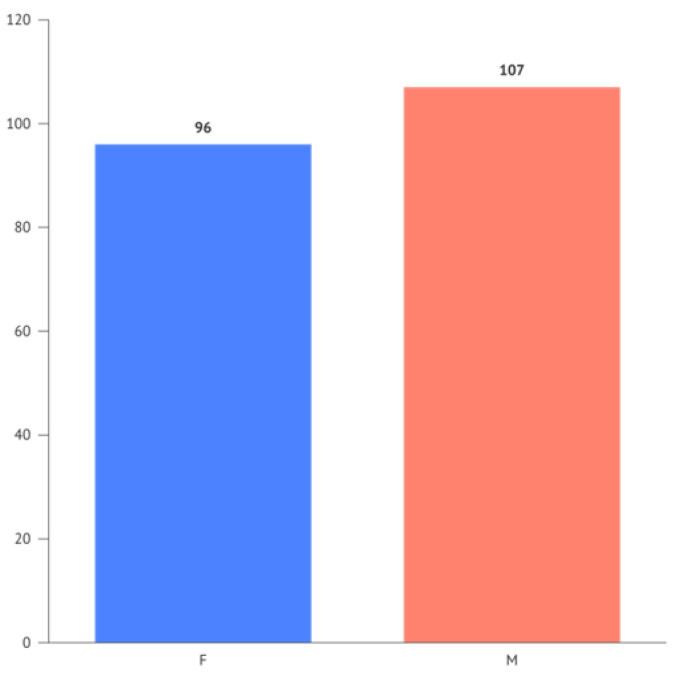
Bar chart of the frequency of periodontitis divided by gender.

The frequency of periodontitis divided by gender is illustrated in Figure 3.

Correlations between numerical variables are presented in Table 2.

The collected data allowed us to statistically assess the correlation between age, perceived stress, PCR, and GBI through Poisson regression analysis. Overall, the analysis revealed that “Age” and “Perceived Stress” were significantly associated with periodontitis. However, “GBI” and “PCR” did not show a significant association with periodontitis in Poisson Regression.

**Figure 4:**
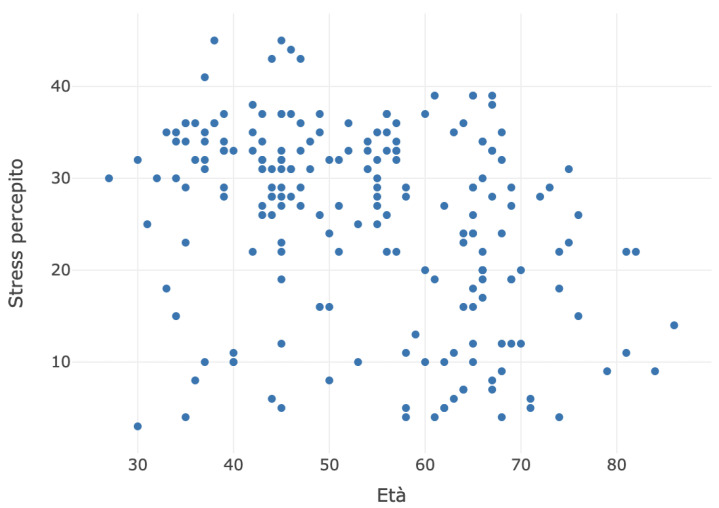
Scatter plot between age and perceived stress.

Additionally, the data collected showed that perceived stress is higher in the age group between 30 and 50 years. The scatter plot between age and perceived stress is depicted in the chart below (Figure 4).

The collected data show that there are no significant differences in clinical periodontal values or psychological scores based on gender. The average of numerical variables for each category of the categorical variable of gender is presented in Table 3.

**Figure 5:**
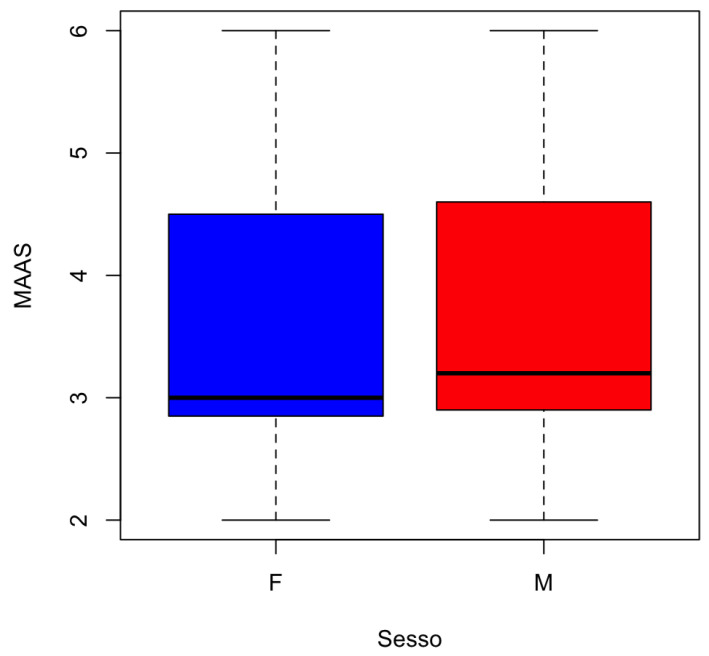
Boxplot for the numeric variable MAAS divided by the categorical variable Gender.

The distribution of MAAS by gender is represented in Figure 5.

The data on MAAS test scores were evaluated in relation to the presence or absence of a diagnosis of periodontitis using the Welch test.

The test reveals a notable difference in the mean MAAS scores between the two groups divided based on the variable “periodontitis”, as the *p*-value is below 0.05.

The data thus highlight that patients with lower MAAS scores, indicating lower self-awareness, statistically have a higher prevalence of periodontitis. Conversely, patients with higher levels of mindfulness statistically exhibit better periodontal health.

The linear regression analysis looked at how Periodontitis (the response variable) is related to several predictor variables (GBI, Age, Perceived Stress, PCR). The residuals, showing the difference between observed and predicted values, ranged from −1.04069 to 0.91313. The estimated coefficients for the predictors varied, with significant values for Age and GBI. The T values indicated significance, especially for Age and Perceived Stress. The residual standard error was 0.3692. The multiple R-squared of 0.3418 means the model explains 34.18% of the variance, with an adjusted R-squared of 0.3285. The F-statistic of 25.71, with a *p*-value less than 2.2 × 10^−16^, shows that the overall model is statistically significant. In this study sample, periodontitis is statistically linked with all the variables studied, especially with increasing age and GBI.

**Figure 6:**
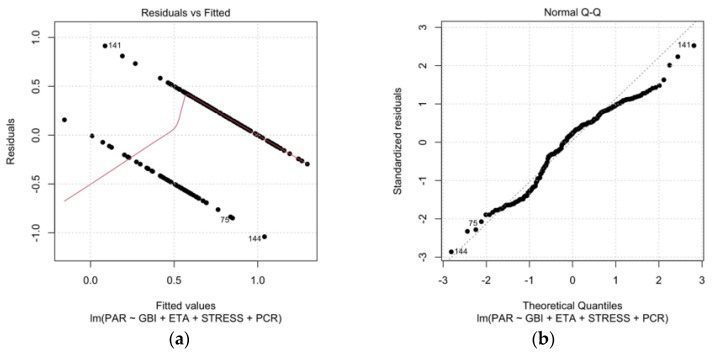
Residual plots of linear regression. (**a**) Residual plot. (**b**) Normalized residuals plot.

All data have been represented in Figure 6a,b.

The Pearson correlation test was used to examine the relationship between Periodontitis and PSS in our dataset. The *p*-value from this test was 0.01666, indicating the likelihood of obtaining a correlation as strong as observed by chance. A *p*-value below the common significance level suggests that the correlation is statistically significant. However, our analysis showed no significant positive correlation between Periodontitis and Perceived Stress. In this study, high levels of perceived stress were found in both patients with periodontitis and in those without it.

**Figure 7:**
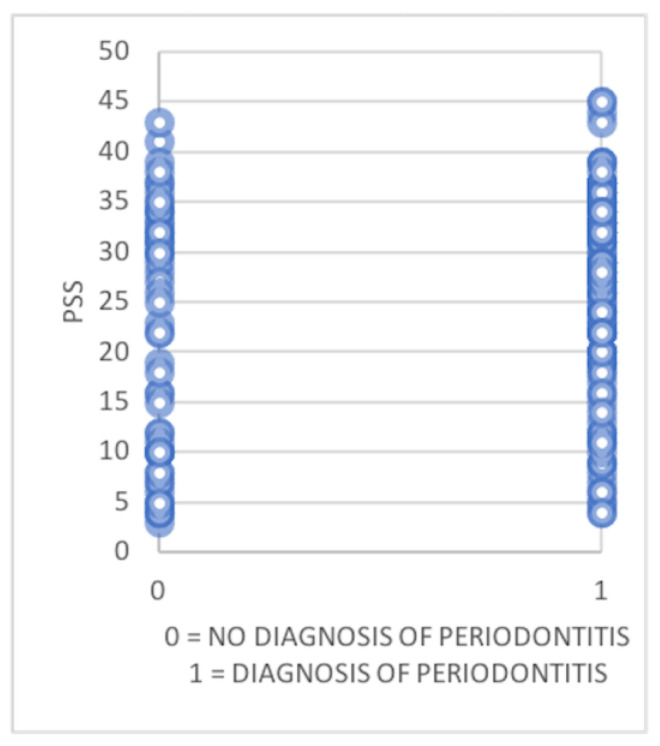
Scatter plot representing PSS values among patients with and without periodontitis.

The Figure 7 graph represents all the described data.

**Figure 8:**
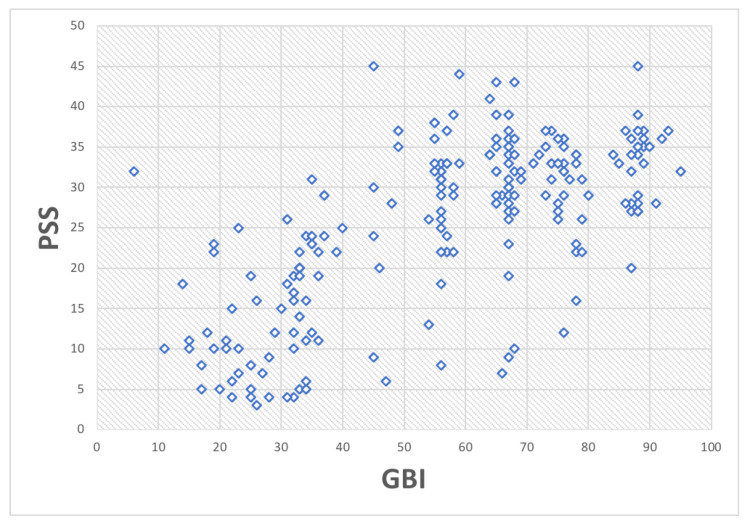
Scatter plot representing the association between GBI and PSS.

A Pearson correlation test was conducted to explore the relationship between the GBI and Perceived Stress in our dataset. The *t*-value, representing the *t*-test statistic for this correlation, was 12.986. With 201 degrees of freedom, our analysis had considerable flexibility. The *p*-value was extremely low, indicating a highly significant correlation. The alternative hypothesis—that the true correlation between these variables is not zero—was supported by our results. The 95% confidence interval for the correlation was between 0.5928944 and 0.7439638, suggesting a high level of certainty in the strength of the correlation. The Pearson correlation estimate was 0.6754551, indicating a moderately strong positive correlation between GBI and Perceived Stress in our dataset. All the data are depicted in Figure 8.

The Pearson correlation test was conducted to explore the relationship between the GBI and MAAS variables within our dataset. As for sample estimates, the Pearson correlation estimator yielded a value of −0.7487295. This indicates a moderately strong negative correlation between GBI and MAAS within our dataset, suggesting an inverse relationship between the two variables.

The Pearson correlation test was used within our dataset to examine the relationship between the PCR and Perceived Stress variables. As for sample estimates, the Pearson correlation estimator was determined to be −0.3738504. This suggests a moderate negative correlation between PCR and perceived stress within our dataset, indicating an inverse relationship between the two variables.

We performed a Pearson correlation test to examine the relationship between the PCR and MAAS variables in our dataset. The 95% confidence interval ranged from 0.2259032 to 0.4676427, suggesting that the true correlation likely falls within this range. The Pearson correlation estimate was 0.3526427, indicating a moderate positive correlation between PCR and MAAS in our dataset. This means that an increase in one variable tends to be associated with an increase in the other.

## 4. Discussion

A wealth of previous research provides strong evidence for a significant correlation between oral health status and a range of chronic diseases that cause inflammatory imbalances [53,54,55,56]. This research forms a pivotal component of a comprehensive exploration into inflammatory pathologies impacting oral health and highlighted the need to assess patients’ emotional and psychological status.

As indicated by the statistical analysis conducted on the population sample within this clinical investigation, a correlation between emotional stress and periodontal health is observed. A significant correlation has been found between perceived stress values and gingival bleeding. Certain clinical studies furnish clinical data assessing the inflammatory molecular constituents among individuals experiencing stress. Two research studies corroborated that these individuals exhibit heightened levels of salivary cortisol and inflammatory cytokines, including IL-1beta and IL-6, in their serum and crevicular fluid, ultimately resulting in gingival inflammation [57].

Recent studies have shown that when a person experiences stress, their body produces corticotropin-releasing hormone (CRH), which can stimulate gingival mast cells. This, in turn, can cause the mast cells to release pro-inflammatory molecules along with other neuropeptides and cytokines, ultimately leading to periodontitis [58,59].

In another recent study, an analysis of salivary chemical components in 117 patients was conducted. Biomarkers with the potential for disease severity categorization include salivary cortisol levels and chromogranin A. Among patients diagnosed with gingivitis and periodontitis, cortisol levels exceeding the normal range and an imbalanced cortisol/DHEA ratio are associated with psychological stress. These biomarkers are significant predictors for detecting and monitoring stress levels in affected patients [60,61].

Variations in the diagnosis of periodontitis across these studies are significant, with some focusing solely on clinical attachment level and others exclusively on probing depth as parameters to delineate groups with and without the disease. Meanwhile, some studies classify periodontitis based on changes in probing depth or clinical attachment level [58,59,60,61].

Additionally, given this diversity in classification criteria, our study aimed to evaluate three approaches to defining periodontitis. While previous studies commonly relied on a single clinical parameter as a surrogate for defining periodontitis, our study investigated three distinct outcome definitions to mitigate the risk of misdiagnosis. We employed the Periodontal Screening and Recording (PSR) score and Gingival Bleeding Index (GBI) to establish a criterion for defining gum disease. Although these clinical data have not been previously utilized in studies on this topic, they have been employed in research associating periodontal infection with systemic diseases and conditions. Furthermore, it is essential to emphasize the importance of conducting a comprehensive periodontal clinical examination, which includes evaluating six sites on each tooth. Previous studies that did not assess all six sites per tooth tend to either underestimate or overestimate the presence of periodontitis, thus increasing the likelihood of misclassification.

In addition to age, the frequency of stress and periodontitis is influenced by gender, with stress being more prevalent among females. Men and women tend to exhibit different psychological and biological responses to stress. Women often face an overload of societal expectations, including personal, biological, hormonal, sexual, and social demands, in addition to career pressures. In contrast, periodontitis is more prevalent among men, with some studies suggesting a possible association between sex hormones, particularly high levels of testosterone, and the disease [62].

There is evidence that the alteration of the immune system, caused by stress, may be another factor contributing to the presence of gingival bleeding, even in patients not affected by periodontitis [63]. As highlighted by the results of this study, even patients with low PCR values, without periodontal pathology, had higher bleeding indices.

Recent studies can also explain this result. An in vitro investigation has highlighted that heightened cortisol levels might contribute to the accelerated proliferation of *P. gingivalis*, a prominent instigator of gum disease. Moreover, diminished immune function has emerged as a potential contributor to the exacerbation of gingivitis. Other studies suggest that neuronal cells can release pro-inflammatory cytokines and chemokines, thereby intensifying chronic inflammation in the periodontium and compromising immune resilience [64,65].

It is crucial to reflect on the evidence of the results highlighted in our study, particularly on the correlation between GBI, PCR, and perceived stress. However, correlation does not imply causality, so we cannot state with certainty that an increase in perceived stress causes an increase in GBI and PCR percentages or vice versa. Other factors may be involved in the relationship between these variables.

It is necessary to highlight some limitations of the study. In the following study, the following parameters were not evaluated: education level, current smoking habit, lung disease, and body mass index. Considering these parameters fundamental for the alteration of gingival bleeding and alteration of periodontal health status, a more thorough evaluation in the analysis of the study group could have provided a more in-depth analysis of the result [66]. Given the multifactorial nature of periodontal pathology, a more comprehensive analysis of patients would undoubtedly have yielded a more precise and accurate assessment of the impact of stress on periodontal health. By delving deeper into patient profiles and considering a broader array of contributing factors, our study could have achieved heightened granularity, providing a nuanced understanding of the intricate interplay between stress and periodontal pathology. Such an in-depth analysis would contribute to refining our comprehension of the complex relationships underlying periodontal diseases, thereby enhancing the depth and reliability of our research findings.

Peaks of stress are also linked to increased unhealthy lifestyles, constituting specific risk factors for periodontitis, such as a greater smoking habit, less physical activity, alcohol and sugar abuse, and less care for oral hygiene at home [67].

The study has additional limitations, particularly in using a stress assessment scale that solely captures perceived stress from the patient’s viewpoint. The study did not incorporate objective stress measures, such as professional psychologists and psychiatrists conducting assessments that evaluate an objective component of patients’ stress. This limitation emphasizes the exclusive dependence on subjective self-reporting for stress evaluation, highlighting the absence of more objective and clinically validated stress metrics in the study’s methodology.

Certain medications can significantly impact periodontal health. For example, immunosuppressive drugs, such as corticosteroids, can weaken the body’s immune response, making it harder to fight off infections, including those leading to periodontal disease. Antihypertensive medications, particularly calcium channel blockers, may cause gingival overgrowth, complicating oral hygiene and potentially leading to periodontal issues. Additionally, medications that induce xerostomia (dry mouth), like certain antidepressants or antihistamines, reduce saliva flow, which can promote plaque accumulation and increase the risk of periodontal disease. These examples illustrate how medications can influence periodontal status, either by directly affecting gum tissue or by altering conditions that increase susceptibility to periodontal disease [68].

Alternative, natural treatments can offer potential benefits for periodontal health. For example, oil pulling with coconut or sesame oil is believed to reduce plaque and improve gum health. Herbal remedies like green tea and turmeric contain antioxidants and anti-inflammatory properties that may help reduce gum inflammation and prevent periodontitis. Aloe vera is another natural product known for its soothing effects on gums and can assist in healing minor oral irritations. Probiotics are also gaining attention for their potential to balance oral microbiota and reduce harmful bacteria linked to periodontal disease. While promising, these treatments should be seen as complementary to, not substitutes for, traditional dental care and should always be used under professional supervision [69].

In addition to assessing the patient’s perceived stress through psychological evaluation, this study also examined the psychological aspect associated with mindfulness.

It was observed that perceived stress does not exhibit a robust correlation with the occurrence of periodontitis. Conversely, the values of MAAS appear to be pertinent. Consequently, mindfulness emerges as a variable worthy of assessment in patients with periodontitis. One of the primary benefits of mindfulness is its capacity to mitigate stress. Effective stress management through mindfulness may thus contribute to reducing inflammation. Mindfulness can also foster a more mindful approach to eating. Awareness of one’s eating patterns and sensations of hunger and satiety can aid in healthier dietary choices, contributing to oral health. Mindfulness practices can help individuals become aware of behaviors related to muscle tension and bruxism, assisting in developing strategies to relax the jaw muscles and alleviate associated muscle tension. Furthermore, patients with high levels of mindfulness tend to take better care of themselves [70].

Integrating mindfulness in health management can offer a holistic approach that could significantly improve our patient’s health management [71]. Further research is needed to fully understand mindfulness’s role in dental practice and develop effective strategies for its successful implementation in patient oral care.

## 5. Conclusions

The statistical analysis showed a significant correlation between Perceived Stress scores, mindfulness levels, and periodontal health indicators. Specifically, higher stress and lower mindfulness were associated with increased Gingival Bleeding Index (GBI) scores. However, this correlation does not imply causation, meaning we cannot confirm whether increased stress leads directly to higher GBI or vice versa. The relationship is likely complex and influenced by other factors requiring further exploration. Given the study’s limitations, quantitative and qualitative assessments of psychological variables may offer new insights into periodontal health management. Yet, more research is needed to integrate the evaluation of a patient’s psychological well-being into periodontitis treatment.

## Figures and Tables

**Table 1:** Basic descriptive statistics.

Age						
	**Min**	**1st Qu.**	**Median**	**Mean**	**3rd Qu.**	**Max**
	27	44	54	53.72	65	86
PCR (%)						
	**Min**	**1st Qu.**	**Median**	**Mean**	**3rd Qu.**	**Max**
	12	25	30	33.73	36.50	89
GBI (%)						
	**Min**	**1st Qu.**	**Median**	**Mean**	**3rd Qu.**	**Max**
	6	34	58	56.43	74.50	95
PSR						
	**Min**	**1st Qu.**	**Median**	**Mean**	**3rd Qu.**	**Max**
	1	2	3	3.079	4	4
PSS						
	**Min**	**1st Qu.**	**Median**	**Mean**	**3rd Qu.**	**Max**
	3	18.50	28	25.35	33	45
MAAS						
	**Min**	**1st Qu.**	**Median**	**Mean**	**3rd Qu.**	**Max**
	2	2.9	3	3.564	4.5	6

**Table 2:** Correlations between numerical variables.

Variables	Age	PCR (%)	GBI (%)	PSR	PSS	MAAS
**Age**	1	0.3982293	−0.2019248	0.4480947	−0.3327279	0.2956923
**PCR (%)**	0.3982293	1	−0.1309450	0.2511429	−0.3738504	0.3526427
**GBI (%)**	−0.2019248	−0.1309450	1	0.1642705	0.6754551	−0.7487295
**PSR**	0.4480947	0.2511429	0.1642705	1	0.1434456	−0.1646394
**PSS**	−0.3327279	−0.3738504	0.6754551	0.1434456	1	−0.8450251
**MAAS**	0.2956923	0.3526427	−0.7487295	−0.1646394	−0.8450251	1

**Table 3:** Average of numerical variables for each category of the categorical variable of gender.

Gender	Age	PCR (%)	GBI (%)	PSR	PSS	MAAS
F	51.58333	32.09375	56.90625	3.010417	26.27083	3.496875
M	55.63551	35.19626	56.00935	3.140187	24.53271	3.623364

## Data Availability

The raw data supporting the conclusions of this article will be made available by the authors on request.
